# A new lymphography protocol and interpretation principles based on functional lymphatic anatomy in lower limb lymphedema

**DOI:** 10.1007/s12565-023-00754-2

**Published:** 2023-12-22

**Authors:** Akira Shinaoka

**Affiliations:** Department of Lymphatics and Edematology, Okayama University Graduate School of Medicine, Dentistry and Pharmaceutical Science, Okayama, Japan

**Keywords:** Lymphatics, Lymphatic vessel, Anatomy, Lymphedema, Lymphography, Lymphoscintigraphy

## Abstract

Indirect lymphatic system imaging is essential for diagnosing lymphatic diseases. The basic methodology involves intradermal or subcutaneous injection of a contrast agent into the surrounding lymphatic capillary, and the flow of the contrast agent is identified using a detector. Many contrast agents that use near-infrared dye, including indocyanine green (ICG) fluorescent lymphography, are available. ICG is rapidly spreading as a convenient and safe lymphedema diagnostic method, because it does not involve radiation exposure, and the imaging equipment is more compact than other devices. The lymphatic system is a semi-open circulatory system with numerous lymphatic capillaries acting as blind ends. Anatomical information on the injection site and observation of specific lymphatic vessels and nodes is important. However, this anatomical information is lacking. Recent reports suggest that ICG fluorescent lymphography can be applied to cadavers in the same manner as living bodies. Furthermore, these reports have demonstrated the functional aspects of the capillary lymph vessel networks as well as their relationship with lymphatic vessels and lymph nodes. This review article describes the historical progression from the old to the new functional lymphatic anatomy and introduces a new functional lymphography technique for the lower limbs.

## Introduction: past lymphatic anatomy and lymphatic imaging

Many anatomists have studied the gross anatomy of the lower limb lymphatic system since its discovery in the 1600s. The detailed and artistic drawings of Italian anatomist Paolo Mascagni became the basis of the lymphatic anatomy landmarks (Mascagni. 1787). Around the 1800–1900s, French anatomist Marie Philibert Constant Sappey and his students compiled nearly 100 years of work, collecting anatomical and systematic data. This work included the classification and anatomical details of the collective lymphatic vessels, including their course and position (Sapppey 1874). This information currently remains the basis for lymphatic anatomy. In the 1900s, Japanese anatomists Buntrou Adachi and Takusaburou Kihara described the detailed anatomy of minor regions, including the deep lymphatics (Adachi et al. 1953; Kutsuna 1968). In recent years, there has been growing interest in studying the lymphatic territory of the skin due to the widespread use of lymphatic drainage techniques for lymphedema. Stefan Kubik was the first to describe the relationship between each collecting lymphatic vessel and the lymphatic territories of the skin (Kubik 1969, 1972, 1973, 1980, 1975; Kubik and Manestar 1995). Until the 2000s, the summary of macroscopic lymphatic anatomy had been completed. However, detailed information was lacking regarding the classification of lymphatic vessels and nodes, the appearance rate of each vessel as well as node, and the anomaly rates. Furthermore, the functional relation between lymphatic capillaries and vessels with the lymph nodes (LNs) in the lower extremities was unknown as lymphatic vessel dissection was a time-consuming process, and conducting the study with multiple cadavers was difficult (Suami et al. 2005; Schacht et al. 2009; Yamazaki et al. 2013).

Lymphatic system imaging has only recently become possible in patients. Lymphatic system visualization can be done via direct methods (Jacobson and Johhanson 1959), wherein a contrast agent is injected directly into the collecting lymph vessels using needle cannulation. Additionally, indirect methods (Witte et al. 2000) involve injecting contrast near the lymphatic capillary lymph vessels in the subcutaneous tissue, which is indirectly taken up by the collecting lymph vessels. In the 1950s, with nuclear medicine advancements, lymphoscintigraphy expanded using albumin or colloids bound to radioisotopes. These were injected subcutaneously and indirectly taken up by the collecting lymph vessels. Despite the low detector resolution of gamma rays and the radiation exposure, lymphoscintigraphy remains the gold standard for lymphatic imaging worldwide. Fluorescent lymphography using the near-infrared dye indocyanine green (ICG) was recently developed in Japan and is spreading worldwide due to its high resolution and limited radiation exposure (Kitai et al 2005; Unno et al. 2008; Akita et al 2020, 2022).

## Functional lymphatic anatomy

The lymphatic system is a semi-open system of vessels, and lymphography can only visualize the lymphatic system that has taken up the contrast agents. Therefore, it is necessary to identify the independent lymphatic units composed of skin lymphatic capillary territory, collecting vessels, and LNs to be evaluated in lymphography. However, several lymph nodes are in the lower extremities, including the groin and below the knee. Since each lymph node is connected to numerous lymphatic vessels, the number and details of independent lymphatic skin territories in the lower limb are unknown.

**Fig.1:**
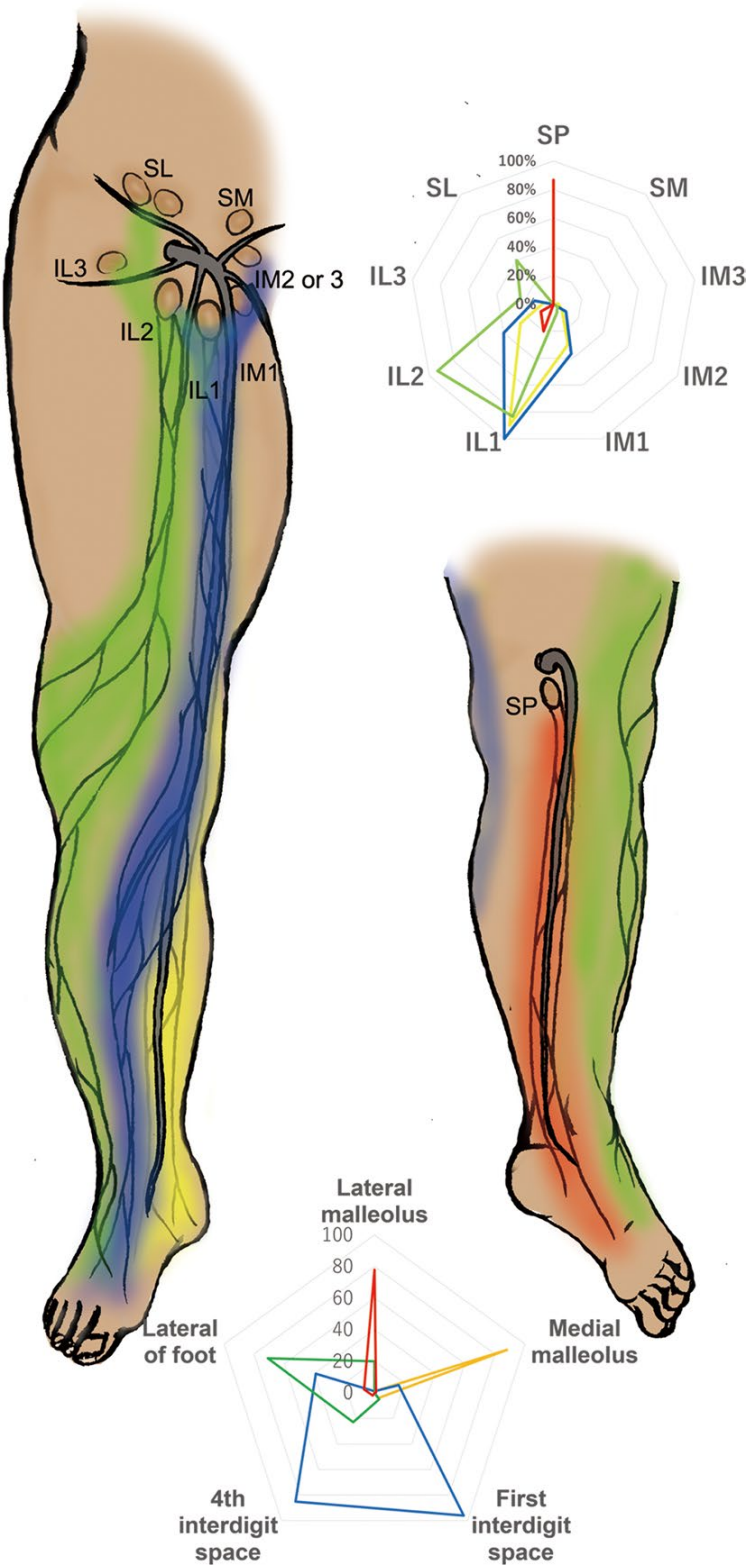
Injection site, lymphatic vessel groups, and lymph nodes (reproduced from Shinaoka et al. 2022). Collecting lymphatic vessel groups (posteromedial [PM]: yellow, posterolateral [PL]: red, anteromedial [AM]: blue, and anterolateral [AL]: green) are demonstrated; the PM runs along the main trunk of the great saphenous vein; the PL runs along the main trunk of the small saphenous vein; and the AL as well as the AM run along the branch of the great saphenous vein. Five injection sites (lateral malleolus, medial malleolus, first interdigit space, 4th interdigit space, and lateral foot) and each lymphatic vessel group were visualized, as demonstrated in the radar chart (below). When ICG was injected from each lymphatic group, it reached the lymph nodes as revealed in the upper radar chart (upper): *IM* inframedial, *IL* infralateral, *SM* supramedial, *SL* supralateral, *SP* superficial popliteal

**Fig. 2:**
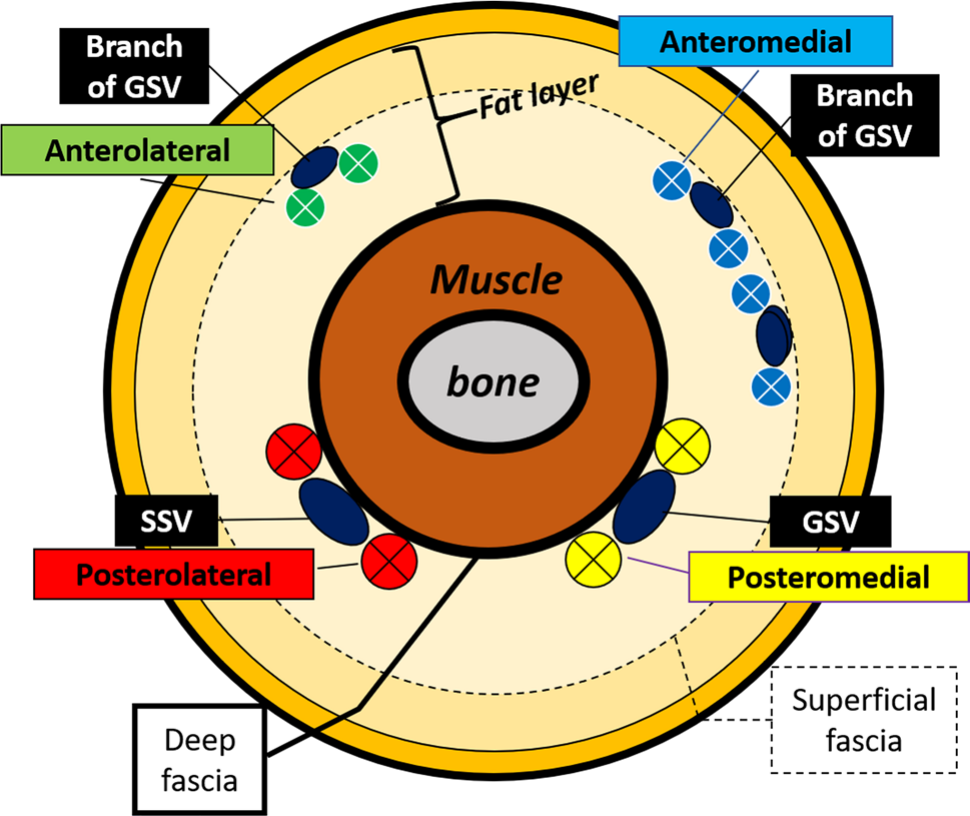
Relationship between the lymphatic vessel groups and veins according to the transverse section of the lower leg. The PL is associated with the main trunk of the small saphenous vein, the PM with the main trunk of the great saphenous vein, and the AM with a branch of the great saphenous vein, *SSV* small saphenous vein, *GSV* great saphenous vein. Posteromedial [PM]: yellow, posterolateral (PL): red, anteromedial [AM]: blue, and anterolateral [AL]: green

Thus, ICG fluorescent lymphography was initially applied to lower extremity cadaver studies to shorten dissection time, elucidate the functional network aspects of lymphatic capillaries, and establish their relationship with the lymphatic vessels (Shinaoka et al. 2018a, b). The study demonstrated that the lymphatic system from the lower extremity periphery has four independent pathways with different anatomical features: anterolateral (AL), anteromedial (AM), posterolateral (PL), and posteromedial (PM) (Shinaoka et al. 2019) (Fig. 1). Of the four pathways, PL and PM were the main pathways that traveled to the main trunk of the small and great saphenous veins, respectively. However, the AM and AL pathways were minor, traveling to the branching veins of the great saphenous vein (Fig. 2). Additionally, the skin areas were identified where the four lymphatic systems were distributed. In the second study, Computed tomography (CT) lymphangiography demonstrated the close relation of the four lymphatic vessel groups with two lymph nodes in the inguinal region and one in the popliteal (Shinaoka et al. 2020).

**Fig. 3:**
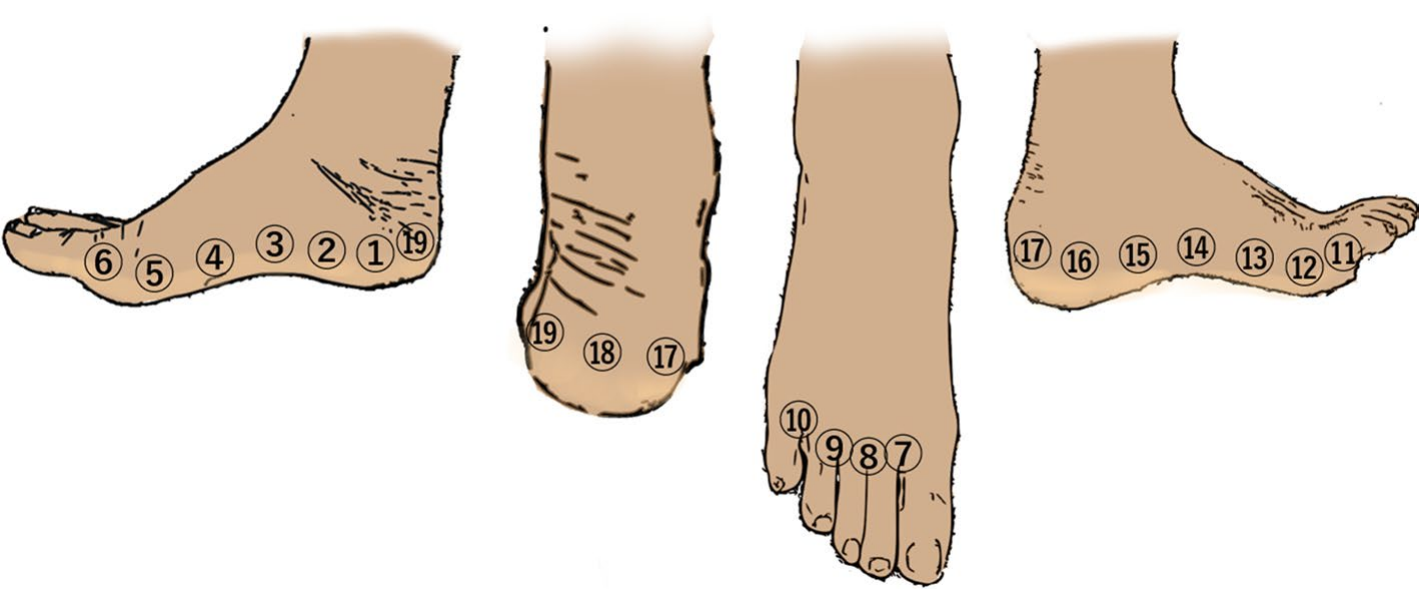
Recommended injection sites. Nineteen injection sites have been identified. All of these injection sites are located at the border between the dorsal and plantar feet, while the most recommended injection sites are just below the medial malleolus for posteromedial (site 1), just below the lateral malleolus for posterolateral (site 16), the first interdigit space for anteromedial (site 7), and the midpoint of the external popliteal and head fifth metatarsal bone for anterolateral (site 14)

In conclusion, these two cadaveric lower extremity studies revealed the functional aspects of each unit composed of lymphatic capillaries, collecting vessels, and regional LNs. Furthermore, these results led to the establishment of a recommended injection site for evaluating collecting vessels and LNs of the lower extremity. The combination of four injection points (Fig. 3: sites 1 for PM, 7 for AM, 14 for AL, and 16 for PL) can be considered tracer injection sites for mapping all four lymphatic vessel groups. Additionally, three injection points (Fig. 3: sites 7, 14, and 16) were used for mapping regional LNs in the lower extremity.

## Methodology of ICG lymphography based on lymphatic anatomy

**Fig. 4:**
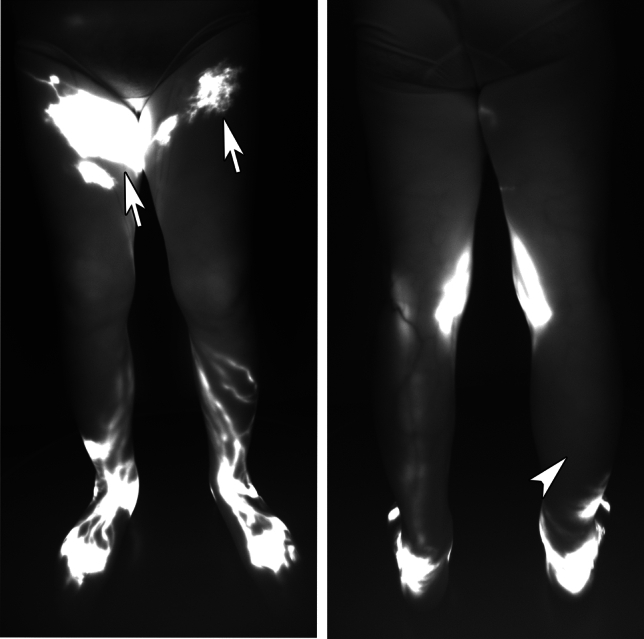
Panoramic view of indocyanine green lymphography. Front (left) view and back (right) view. The dermal backflows (arrow) were observed in the thigh, while the posterolateral pathway in the right leg was missing (arrow head).

The purpose of lymphography for patients is to determine which lymphatic pathways remain functional as well as which are obstructed, identify lymphatic flow leakage locations into the skin [dermal backflow (DB)], and trace the final lymph flow destination (Shinaoka et al. 2017: Suami et al. 2018; Yamamoto et al. 2011) (Fig. 4). Thus, an accurate diagnosis requires evaluation of the whole lymphatic system in the lower limb.

In the lower extremities, the first interdigital space is the most common tracer injection site (Bourgeois 2007; Notohamiprodjo et al. 2012). However, injection sites using the second interdigital space (Gloviczki et al. 1989) and multiple interdigital spaces have also been reported (Szuba et al. 2003; Lohrmann et al. 2006; Maegawa et al. 2010). According to the latest results (Shinaoka et al. 2019; Shinaoka et al. 2020), toe web space injections only demonstrated the AM pathway while missing the other three pathways. Thus, three additional injection sites are required for analyzing the PM, AL, and PL groups. Lymphangiography textbooks recognize the PL and AL pathways as independent pathways from the AM pathway and recommend cannulating and injecting these three pathways separately (Kimonth. 1952).

If the DB expands immediately after injection, it hides the remaining lymphatic vessels, making ascertainment difficult. The patient was asked to rest after the injection to prevent obscuring the remaining vessels, and only the injection site was massaged with the fingertips. After the ICG has been taken up by the lymphatic vessels, gentle massage along the lymphatic vessels with the fingers was performed to aid the ICG ascent to the lymph nodes, allowing us to evaluate the entire lymphatic flow. However, if the DB only begins to appear, stop massaging the area and focus on stimulating other lymphatic vessels as much as possible to obtain an overall picture. Subsequently, mapping is performed by massaging the lymphatic vessels where the DBs appear.

Exercise stress is usually added to accurately assess the DB appearance and extent (Engeset et al. 1977; Gloviczki et al. 1989; Havas et al. 1997). As DB often appears near the inguinal area, the examination cannot be completed without ICG flow to this area. However, due to the slow flow of lymphatic fluid, 15 min of walking exercise has been reported to be necessary for promoting lymphatic flow and allowing ICG movement to the inguinal region (Matsumoto et al. 2019). The order of ICG injection is also important as the primary PM and PL lymphatic vessels run over the deep fascia, making it difficult to observe them if the AL and AM, which extend into the shallow subcutaneous layers, are contrasted first. Due to this, ICG is first injected to target the PM and PL (Fig. 3: site 1 and 16) to confirm the overall flow and subsequently injected to target the AL and AM (Fig. 3: site 7 and 14). Confirming the presence of PM and PL is crucial as they tend to be deficient in the early lymphedema stages, ensuring an accurate diagnosis.

## Interpretation of ICG lymphography based on lymphatic anatomy

It is necessary to confirm the presence and extent of DB (Yamamoto et al. 2011) and the lymphatic pathway defects (Kimonth. 1952) to diagnose lymphedema and assess its severity. The presence or absence of DB is the most important criterion in diagnosing lymphedema. DB commonly appears in the inguinal region but may also appear peripherally, solely at the injection site, or within one lymphatic pathway. Therefore, it is essential to initially contrast the entire area and meticulously observe every detail. If edema other than lymphedema is present at the injection site, ICG may spread subcutaneously, potentially causing a vague diagnosis. In such cases, the diagnosis of DB is confirmed only when there is an observation of ICG spreading into the capillary lymphatics under high power magnification.

**Fig. 5:**
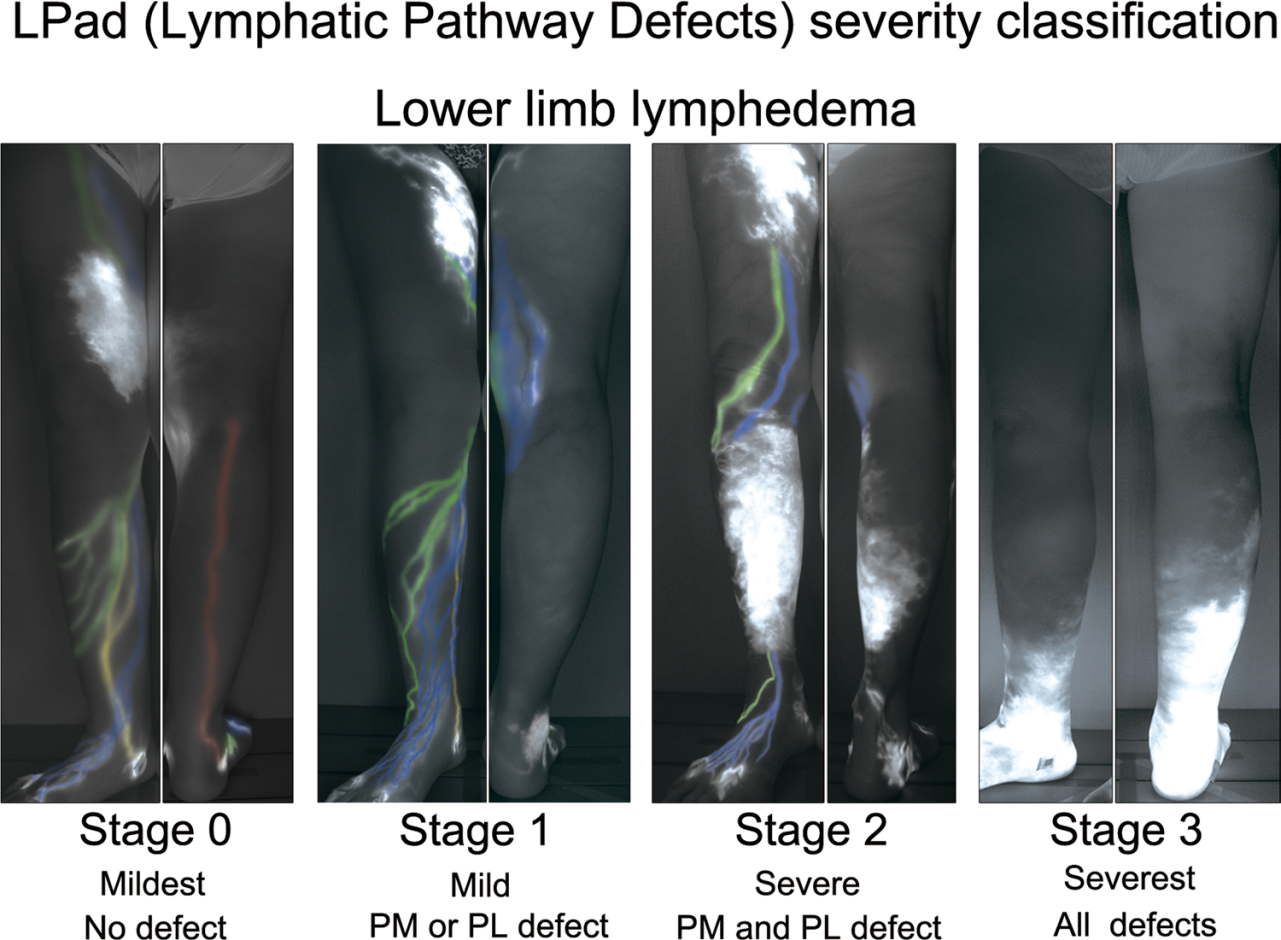
The lymphatic pathway defect (LPad) severity classification in lower extremity lymphedema. (Reproduced from Shinaoka et al. 2022). Indocyanine green lymphographic images of the four legs with lymphedema depicting four colored lymphatic vessel groups and dermal backflow: anteromedial (blue), anterolateral (green), posteromedial (yellow), and posterolateral (red). Double defects in the posteromedial and posterolateral groups were more severe than the single defects of these two groups. Defects in all lymphatic groups, including the anteromedial and anterolateral groups, were more severe than double defects. Previous reports have revealed that in the terminal stage of leg lymphedema, all lymphatic collecting vessels in the legs are lost

While there is a relationship between the appearance of DB and lymphatic pathway defects, these two often occur independently and are separate findings. Since the four lymphatic pathways are almost always present in the normal state, these defects may indicate changes in the lymphatic system. Previous studies have reported that lymphatic pathway defects are strongly associated with lymphedema severity (Shinaoka et al. 2022). In normal-to-mild lymphedema, there is no lymphatic pathway defect. When the PL or PM is defective, the severity increases by one step to mild lymphedema. When the PM and PL are defective, the severity increases by another step. AM and AL often remain intact in severe lymphedema; however, when AM and AL are deficient, lymphedema progresses to the most severe type. Given these results, a severity classification was established to evaluate lymphedema, known as the Lymphatic Pathway defect (LPad) (Fig. 5).

## Conclusion

A new functional lymphatic anatomy was added to the previous one, leading to the establishment of a lymphography methodology in the lower extremity from an anatomical perspective. The limitations of the functional lymphatic anatomy include the lack of information about deep lymphatics. Accordingly, additional study about anatomy of deep lymphatics will be needed for further understanding of lymphedema pathology

## Data Availability

The data that support the findings of this study are available from the corresponding author, [A.S.], upon reasonable request.
